# Inhaled Halogen‐Induced Oxidative Renal Damage and Dysfunction: A Lung Heart Kidney Axis

**DOI:** 10.1002/cph4.70096

**Published:** 2026-01-06

**Authors:** Juan Xavier Masjoan Juncos, Ahmed Zaky, Wesam Nasser, Amber J. Johns, Iram Zafar, Gajanan R. Jadhav, Jeremy B. Foote, Aftab Ahmad, Anupam Agarwal, Shama Ahmad

**Affiliations:** ^1^ Department of Anesthesiology and Perioperative Medicine University of Alabama at Birmingham Birmingham Alabama USA; ^2^ Comparative Pathology Laboratory University of Alabama at Birmingham Birmingham Alabama USA; ^3^ Division of Nephrology Department of Medicine University of Alabama at Birmingham Birmingham Alabama USA

**Keywords:** halogen, inhaled, injury, organ damage, oxidative stress, renal, rodent

## Abstract

Acute exposure to halogen gases causes extensive injury to the lungs and heart that may be fatal. To evaluate secondary renal complications subsequent to pulmonary and cardiac dysfunction, rats were exposed to bromine and chlorine, and their renal function and injury biomarkers were assessed post exposure. Bromine or chlorine caused a significant increase in arterial blood creatinine and urea nitrogen (BUN) suggesting acute renal stress. Rats exposed to either of these halogens also exhibited increased total protein, albumin, and retinol binding protein 4 (RBP4) in the urine indicating significant kidney damage. Significant increases in kidney injury markers such as kidney injury molecule‐1 (KIM‐1), neutrophil gelatinase‐associated lipocalin (NGAL), and osteopontin were observed in the urine and kidney tissues in addition to structural changes further demonstrating tubular damage and cast formation. Hemodynamic parameters such as the mean arterial blood pressure (MAP) and renal vascular resistance (RVR) increased, while the renal artery diameter and renal blood flow decreased significantly. Observation after 4 weeks, following bromine exposure, demonstrated increased collagen volume in the interstitium and the glomerulus, and increased fibrosis characteristic of the progression of acute kidney injury (AKI) to chronic kidney disease (CKD). The renal tissues showed increased myeloperoxidase and plasma oxidized low‐density lipoproteins further indicating oxidative stress in the halogen exposed animals. Kidneys are highly susceptible to oxidative stress which causes cell damage, death, and renal dysfunction leading to AKI. Clinically, these data suggest that victims of halogen exposure are at increased risk of cardiovascular events as well as renal dysfunction and AKI.

## Graphical abstract

**Mechanisms of lung‐heart‐kidney interaction in inducing organ injury after chemical inhalation**. Acute exposure to halogen gases causes extensive lung injury and acute cardiac damage leading to fatalities. Formation of highly reactive halogenated molecules in the pulmonary bed and their circulation resulting in oxidative stress and inflammation was responsible for the toxicity. Kidneys are highly susceptible to oxidative stress, which causes oxidative cell damage, cell death, and renal dysfunction leading to acute kidney injury. This damage was enhanced by reduced perfusion due to the parallel cardiac damage and dysfunction. A vicious cycle of secondary organ interaction perpetuates the injury and enhances the toxicity (Related but previously published work is depicted in the gray squares).

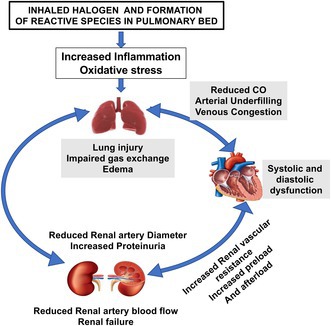

## 1 Introduction

Exposure to halogen gases like chlorine (Cl_2_) and bromine (Br_2_) causes extensive lung injury that may lead to acute respiratory distress syndrome (ARDS) (Lam et al. 2016; Lambert et al. 2017; Lazrak et al. 2015; Leikauf et al. 2012; Summerhill et al. 2017; Radbel et al. 2020; Zellner and Eyer 2020; Zhou et al. 2018). Due to their high reactivity with water, both chlorine and bromine readily produce high concentrations of their hydrolysis products, such as hypohalous and hydrohalic acids (Addis et al. 2021). The high reactivity of these products with organic compounds is the primary cause of toxicity and often correlates with organ injury (Addis et al. 2021; Juncos et al. 2020). Our work with Cl_2_ and Br_2_ exposure‐induced toxicity provided understanding of basic mediators responsible for both pulmonary as well as cardiac injury and discovered novel pathways to test interventions that not only improve immediate survival but also mitigate long‐term effects on the myocardium that often lead to heart failure. Cardiac effects of Cl_2_ were described primarily in human autopsy reports. A severely dilated right heart was observed in victims (that died) of World War I Cl_2_ poisoning. On the other hand, the cardiovascular impact of acute Br_2_ inhalation was found to be severe on site. This was characterized by hypoxemia, hypercapnia, sinus tachycardia, and cardiac arrhythmias that progress to cardiac arrest and circulatory collapse (Makarovsky et al. 2007; Morabia et al. 1986). Acute accidental Br_2_ exposure of a pharmaceutical plant operator caused systemic injuries along with myocardial toxic dystrophia (Liubchenko and Alekseeva 1991). Chronic myocardial degeneration and hypotension was observed in patients that accidentally inhaled Br_2_. This was also replicated in our recent preclinical studies that determined that a single halogen/Br_2_ exposure can cause long‐term cardiac damage, remodeling, and systolic and diastolic dysfunction in the survivors (Juncos et al. 2020; Masjoan Juncos et al. 2021a, 2021b, 2024). Whereas the renal structure and function are frequently studied in acute respiratory distress syndrome (ARDS), less is known about effects of inhaled halogen on the kidneys (Darriverre et al. 2020; Farha and Munguti 2020; Malek et al. 2018; Seeley 2013). Cardiac dysfunction or failure, on the other hand, is known to impact the kidneys through reduction in renal blood flow (Jankowski et al. 2021; Zhang et al. 2023; Zoccali et al. 2024). Administration of halogen‐substituted compounds were shown to cause significant nephrotoxicity (Hong et al. 1996; National Institutes of Health 1996; Valentovic et al. 1992; Muto et al. 1992; Lag et al. 1991). Here we describe a halogen inhalation model in rats where ischemic cardiac injury (Ahmad, Masjoan Juncos, et al. 2019) and AKI occur concurrently and provide a systematic evaluation of the underlying mechanisms.

## 2 Materials and Methods

### 2.1 Animals

Sprague Dawley rats (250–300 g, 8–10 weeks old) were obtained (Inotiv/Envigo), housed, cared for, and used according to approved protocols and University of Alabama at Birmingham (UAB) Institutional Animal Care and Use Committee (IACUC) guidelines. Our prior studies with female rats had revealed similar injury upon high dose halogen gas exposures, and therefore, to minimize redundancy and animal use, only male rats were used (Masjoan Juncos et al. 2021a). Two rats per cage were housed under a 12 h dim light/12 h dark cycle in standard polycarbonate cages with wood chip bedding and nesting enrichment (EnviroPak). Standard diet and tap water were provided ad libitum, and the room temperature was maintained at 70°F with humidity maintained between 40% and 60%. A priori power analysis was performed using ANOVA F‐test to compute sample size. Assuming the group sizes with a power of 95% and α‐error probability of 5% or less, expected mortality, and an effect size of 0.5–0.7, the number of animals required for proposed studies was calculated. Rats were euthanized at the study endpoint in accordance with 2020 American Veterinary Medical Association Panel on Euthanasia Guidelines to allow for specimen collection. For blood and tissue collection, general anesthesia was induced by 5% isoflurane (Piramal Critical Care, Telangana, India) and maintained by 2% isoflurane. After ensuring adequate depth of anesthesia, incisions were made to open the abdominal cavity, bowels were gently displaced, and the abdominal descending aorta was exposed. Blood was collected from the descending aorta using a heparinized syringe with a 25G needle and analyzed for arterial blood gas (ABG) using an EPOC blood gas and electrolyte analyzer (Heska, Loveland, CO). Exsanguination was then performed to euthanize for necropsy, and renal tissues were collected.

### 2.2 In Vivo Cl_2_ & Br_2_ Exposure, Ultrasound, and Hemodynamics

Unanesthetized rats were exposed (whole body) to 600 ppm of either Cl_2_ or Br_2_ (Airgas, Birmingham AL, gas concentrations in balance air was certified within 2% by the manufacturer) for 45 min as previously described (Ahmad, Masjoan Juncos, et al. 2019; Aggarwal et al. 2016; Leustik et al. 2008). Rats were then returned to room air (RA) and monitored continuously for 6 h and then at 24 h and every 24 h after that until 4 days or 4 weeks after exposure depending on time of end point evaluation. Animals for ultrasound and hemodynamic studies were used at 24 h after exposure. All hemodynamics and ultrasound measurements were performed under 2% isoflurane in compressed room air (RA) and body temperature was maintained at 37°C as previously described (Lindsey et al. 2018). Intraventricular pressures were measured using a 1.4‐Fr high fidelity catheter (SPR 671, Millar institute, Huston, TX), and analyzed using AcqKnowledge III software (ACQ 3.2; Biopac Systems, Galeto, CA) as previously described (Ahmad, Masjoan Juncos, et al. 2019). Echo/Doppler data was acquired using Vevo 2100 high resolution ultrasound system with the 21‐MHz MS250 MicroScanTransducer (VisualSonics, Toronto, ON, Canada) as previously described (Zaky et al. 2015). Data obtained from Echo/Doppler were assessed as described in our previous publication (Ahmad, Masjoan Juncos, et al. 2019). Operators were blinded to exposure performed during image collection and analysis.

### 2.3 Tissue Analysis

Kidney tissue was fixed with 70% alcoholic formalin, paraffin embedded and 5 μM sections and slides were prepared from the blocks and stained with hematoxylin and eosin (H&E) or Picro‐Sirius Red (PSR) as previously described (Ahmad, Masjoan Juncos, et al. 2019). Western blotting was performed on kidney tissue lysates with anti‐rat KIM‐1, NGAL (Abcam, Waltham MA), and β‐actin antibodies (Abcam, Waltham MA).

### 2.4 Renal Function and Injury Marker Measurement

Blood was collected from the descending aorta and serum, and plasma were stored at −80°C. Serum creatinine was evaluated utilizing the LC–MS/MS system of the UAB‐UCSD O'Brien Center for Acute Kidney Injury Research as previously described (Curtis et al. 2021). Urine was collected from the bladder, centrifuged to remove debris, and the supernatant was stored at −80°C. Urine was analyzed for total protein and albumin content. Retinol binding protein 4 (RBP4) content of the urine was evaluated using a commercial ELISA kit (Elabscience, Houston TX). A kidney injury panel (Mesoscale Discovery, Rockville MD) was used to measure urinary levels of NGAL, KIM‐1, and osteopontin following instructions provided in the manufacturer's datasheet.

### 2.5 Statistical Analysis

Results are expressed as mean ± standard error of mean (SEM) of 5–6 rats per group. Graphpad Prism version 10.5.0 software (Graphpad, LaJolla, Ca) was used to perform analyses. Student's *t*‐test was used for pairwise comparisons and to determine variance. ANOVA was performed for multiple group comparisons of normalized data followed by Tukey's post hoc test. Statistical significance was considered for *p* values of < 0.05.

## 3 Results

### 3.1 Halogen Gas (Chlorine and Bromine) Exposure Increases Creatinine Content in the Arterial Blood

**FIGURE 1:**
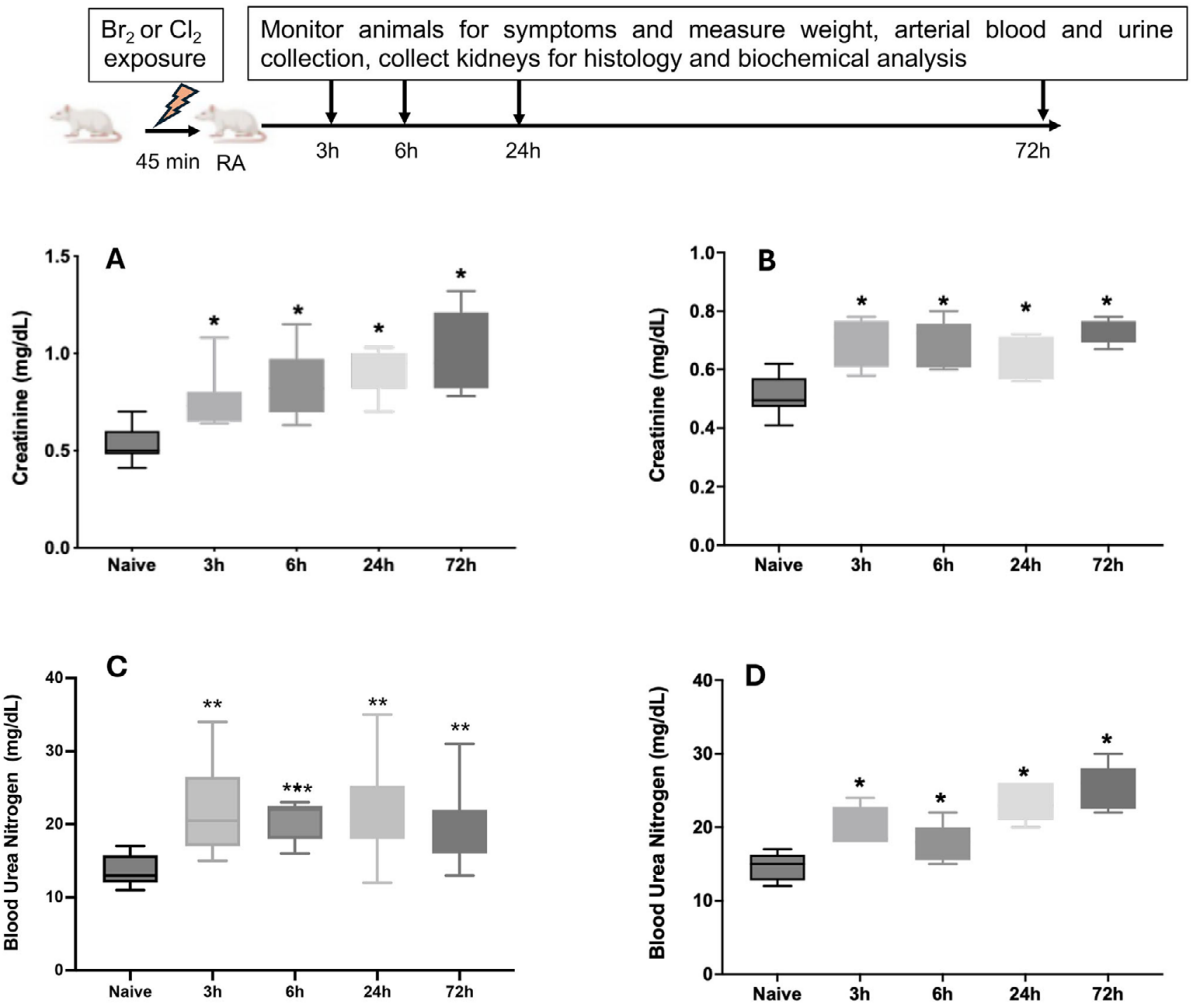
**Exposure to chlorine and bromine causes increased blood creatinine and urea nitrogen content**. Rats were separately exposed to chlorine (600 ppm for 45 min) or bromine (600 ppm for 45 min) and transferred to room air (top panel for schematics). Animals were sacrificed at various time intervals post‐exposure and blood from the descending aorta was collected and analyzed for arterial blood gases (ABG) using a Heska EPOC machine as described in the Methods section. (A) blood creatinine levels of chlorine exposed rats at various time intervals post‐exposure and (B) blood creatinine levels of bromine exposed rats at various time intervals post‐exposure. (C) Blood urea nitrogen (BUN) content of chlorine‐exposed animals. (D) Blood urea nitrogen (BUN) content of bromine‐exposed animals. Values shown are Mean ± SEM (*n* = 6–14). * Indicates *p* < 0.05 as compared to unexposed controls (naives).

Our previously published studies demonstrated that blood sampled from the coronary sinus of chlorine (500 ppm, 30 min) exposed rats had significantly increased creatinine content 3 h after exposure. This increase in creatinine potentially reflects a cardio‐renal interaction besides cardiomyolysis as these rats were undergoing biventricular failure and cardiac contractile dysfunction (Zaky et al. 2015). With arterial blood gas (ABG) and electrolyte analysis using an EPOC/Heska machine we demonstrate that exposure to chlorine (600 ppm for 45 min) and bromine (600 ppm for 45 min) significantly increased the creatinine content of arterial blood collected from the descending aorta at several time intervals (3–72 h) (Figure 1A,B). Bromine exposure caused significantly increased creatinine levels 24 h after exposure in both males and females as shown in Figure S1A. However, serum creatinine of samples from bromine exposed animals, when stored frozen at −80°C and measured by mass spectrometry (LC–MS/MS system) were not elevated (Figure S1B). Blood urea nitrogen (BUN) content was significantly increased (3–72 h) after chlorine or bromine exposure (Figure 1C,D) further indicating renal stress in the exposed animals.

### 3.2 Bromine Exposure Increases Albuminuria and Proinflammatory Kidney Injury Biomarkers in Urine and Kidney Tissues

**FIGURE 2:**
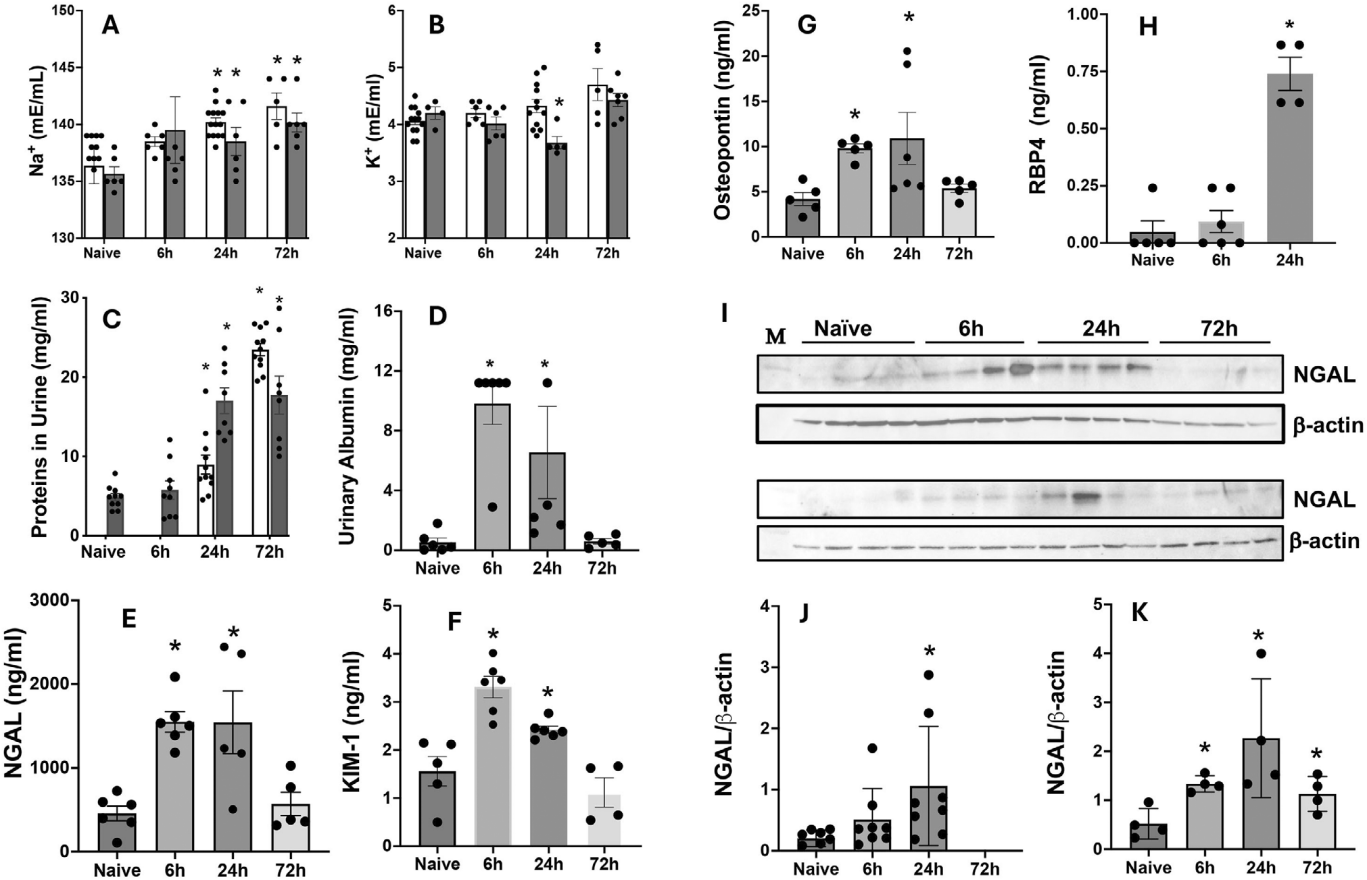
**Chlorine and bromine exposure leads to altered blood ion contents, albuminuria, and increased kidney injury biomarkers.** Chlorine or bromine exposure was performed as described in the Methods section and above and animals were euthanized to collect arterial blood, urine, and kidney tissues. Sodium and potassium ion content were measured in the arterial blood by using a Heska EPOC machine (A and B), where white bars represent values in chlorine and gray bars represent values in bromine. Urine protein content was evaluated to assess renal damage (C), where white bars represent values in chlorine and gray bars represent values in bromine. Urine albumin (D), NGAL (E), KIM1 (F) and osteopontin (G) and RBP4 (H) were evaluated as part of a commercial kidney injury panel kit and an ELISA assay as described in the Methods section. Western blotting was additionally performed to evaluate the injury marker NGAL in the renal tissues (I) where the top panel is for chlorine exposed tissue and the lower panel is for bromine exposed tissue and the densitometric evaluation of the bands with respect to β‐Actin loading control was also performed and shown in the lower panels (J) for chlorine and (K) for bromine. Values shown are Mean ± SEM (*n* = 6–14). * Indicates *p* < 0.05 as compared to unexposed controls (naives).

Blood electrolyte disturbances are surrogates of kidney dysfunction (Marahrens et al. 2023). Arterial blood gas (ABG) analysis of both chlorine‐ and bromine‐exposed animals demonstrated significantly increased sodium at 24 h post‐exposure and remained elevated up to 72 h post‐exposure (Figure 2A). Potassium content decreased by 24 h post exposure but normalized at 72 h in the case of bromine (Figure 2B). Increased urinary protein and albumin are sensitive biomarkers of acute glomerular damage (Bolisetty and Agarwal 2011; Ware et al. 2011). We collected the urine of naïve (unexposed) animals and also that of chlorine or bromine exposed animals at 6, 24, and 72 h post‐exposure and evaluated these samples for total protein and albumin content (Figure 2C,D). Total protein content of the urine from both chlorine and bromine‐exposed animals was elevated at 6 h and stayed significantly elevated up to 72 h post‐exposure. Because rats have a functional ‘proteinuria’ we used albuminuria as a species‐specific protein to assess for acute renal damage. Albumin content was significantly increased in the urine of bromine‐exposed animals at 6 and 24 h after exposure (Figure 2D). Although protein content continued to be elevated at 72 h post‐ exposure, albumin was not detected at that time point. Neutrophil gelatinase associated lipocalin (NGAL) is also known as the ‘renal troponin’ and is detectable in urine and plasma even before creatinine in AKI (Kubrak et al. 2018). Another quantitative kidney injury biomarker is kidney injury marker 1 (KIM1). Urinary NGAL (Figure 2E) and KIM1 (Figure 2F) were significantly increased at 6 and 24 h following bromine exposure. Osteopontin is also a biomarker indicative of supersaturation and crystallization of the urine. Osteopontin is released from the tubules into the circulation upon acute kidney injury and can cause further damage to distant organs such as the lungs (Khamissi et al. 2022). We measured urinary osteopontin levels which were significantly elevated in bromine‐exposed animals at 6 and 24 h post‐exposure (Figure 2G). Urinary retinol binding protein 4 (RBP4) is an index of tubular injury and its elevation in urine reflects renal proximal tubular dysfunction (Ratajczyk et al. 2022). Our study shows that urinary RBP4 was significantly elevated after 24 h in bromine‐exposed animals (Figure 2H). We also assessed the levels of NGAL in the kidney tissues of bromine and chlorine exposed rats by Western blot (Figure 2I). Exposure to the halogen gases caused an increase in NGAL in the kidney tissues (Figure 2I–K). Bromine exposure significantly caused accumulation of both NGAL and KIM 1 in the kidneys by 24 h (Figure S2).

### 3.3 Bromine Exposure Reduces Renal Blood Flow, Narrows Renal Artery Diameter, and Increases Renal Vascular Resistance and Mean Arterial Pressure

**FIGURE 3:**
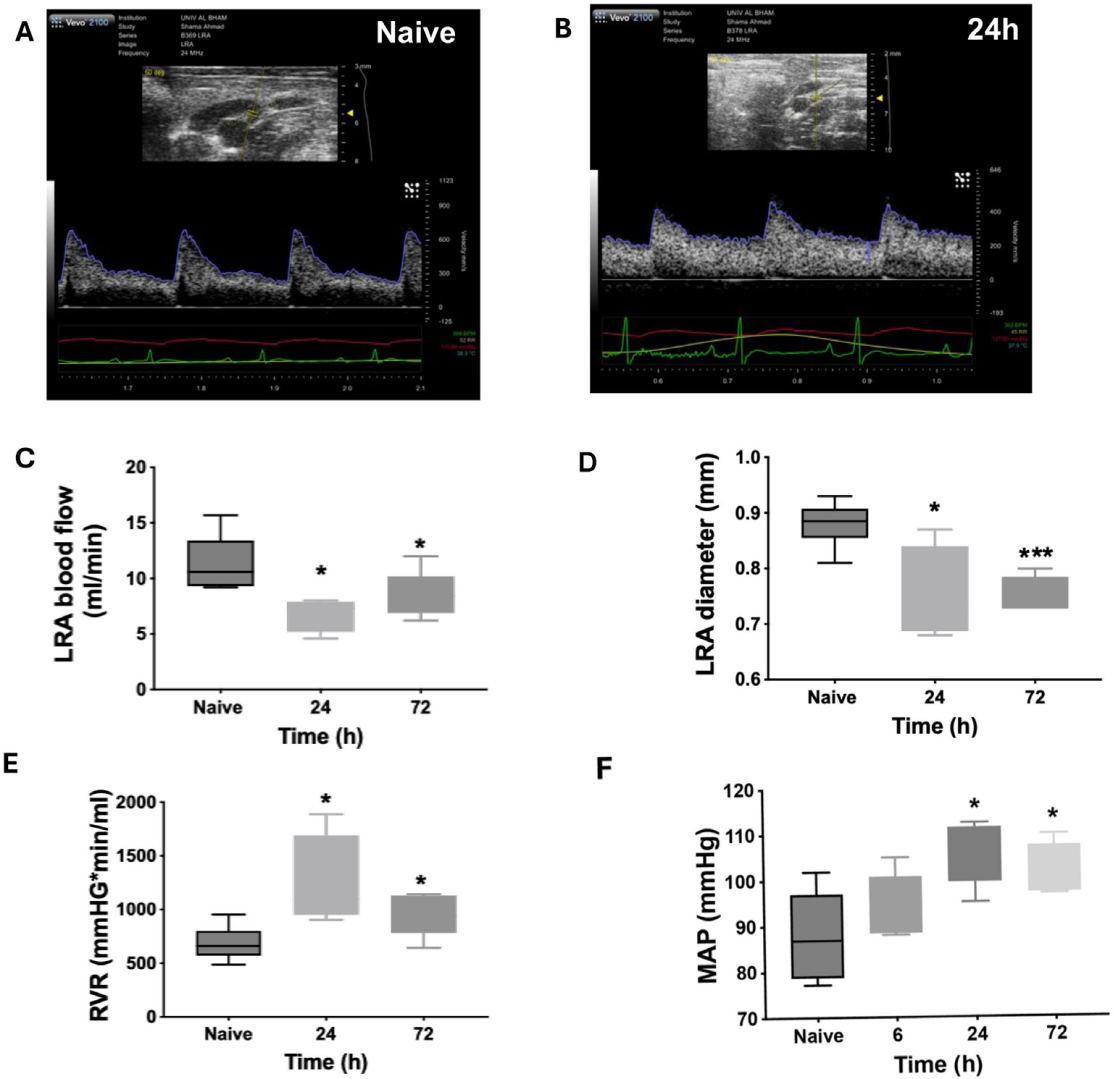
**Bromine exposure reduces blood flow in the renal arteries, narrows renal artery diameter, and increases renal vascular resistance.** High resolution color doppler sonographic imaging was performed on kidneys following bromine exposure and compared to those of the unexposed/naïve animals (panel A and B show representative images of naïve and 24 h post exposure). Pulse wave velocity measurements were used to evaluate renal vascular resistance, RVR (C), blood flow in the left renal artery (LRA) (D), and LRA diameter (E) at 24 h and 72 h after exposure. Hemodynamic measurements were used to evaluate mean arterial pressures (MAP) (F). Data are presented as Mean ± SEM (*n* = 6–14). * Indicates *p* < 0.05 as compared to unexposed controls (naives) and *** indicates *p* < 0.001 as compared to unexposed controls (naives).

To further evaluate the physiological impact of bromine inhalation on the renal system we performed high resolution color doppler sonography on the kidney and the renal arteries (Figure 3A,B). The regulation of renal blood flow is critical for kidney function, body fluid, and electrolyte homeostasis. Bromine exposure significantly reduced blood flow in the left renal artery (Figure 3C). This may be a result of the significantly reduced renal artery diameter as observed in bromine exposed animals 24 h post‐exposure (Figure 3D). Renal vascular resistance was also significantly increased as compared to the unexposed animals 24 h after bromine inhalation (Figure 3E). Our previous studies have reported increased systemic vascular resistance and mean arterial pressure (MAP) after bromine exposure (Masjoan Juncos et al. 2021b; Ahmad, Masjoan Juncos, et al. 2019). Also consistent with our earlier findings, hemodynamic analysis revealed significantly increased MAP at 24 and 72 h following bromine exposure (Figure 3F).

### 3.4 Bromine Inhalation Induces Acute Structural Kidney Damage

**FIGURE 4:**
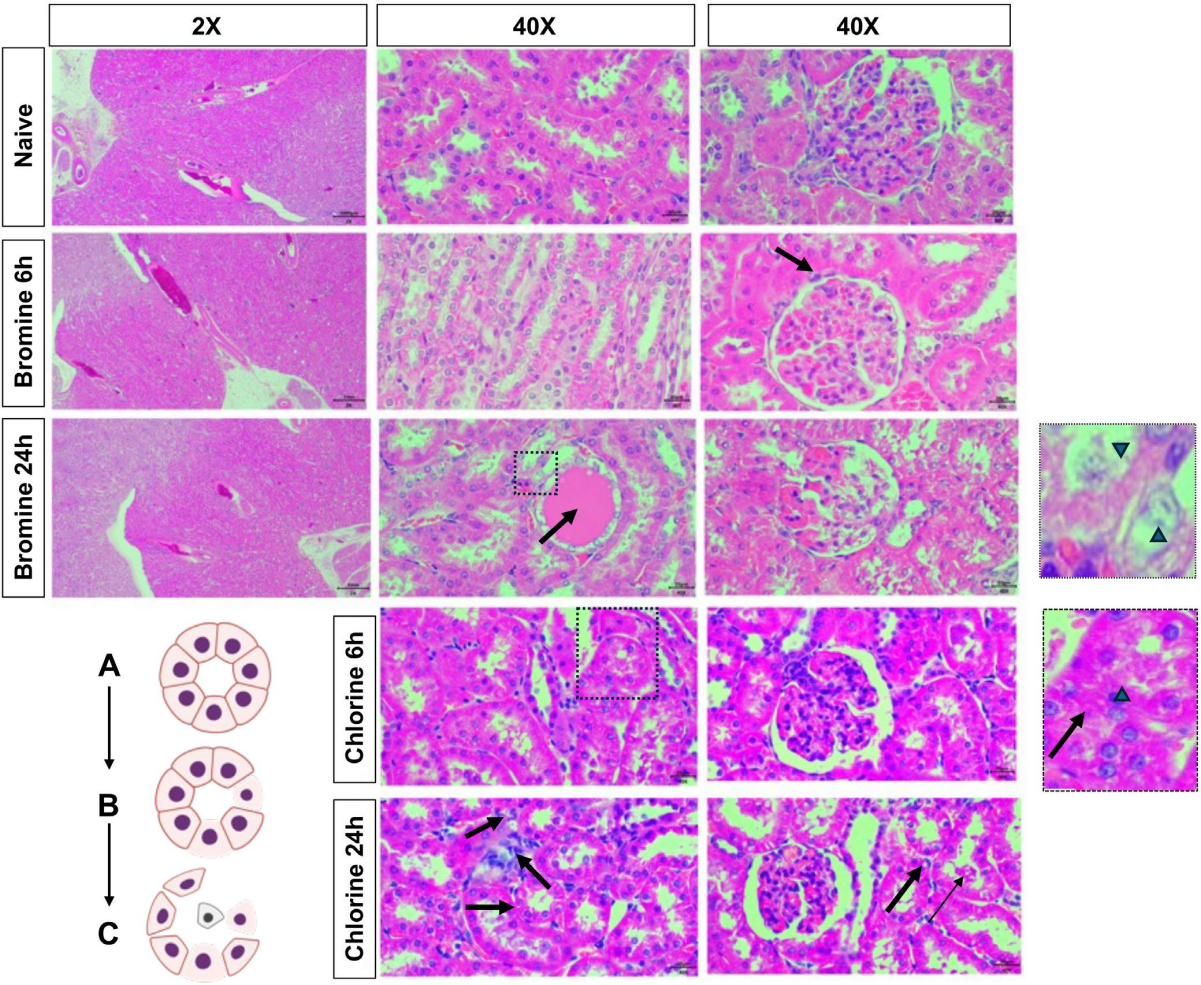
**Chlorine and bromine inhalation induces acute structural kidney damage.** Rats were exposed to chlorine or bromine, returned to room air, and euthanized at 6‐ or 24‐h post‐exposure, as described in Figure 1 and the Methods section. Kidneys were fixed, embedded, sectioned at 5 μm, and stained with hematoxylin and eosin (H&E). The top panel shows representative H&E‐stained kidney sections at 2X or 40X (scale bar = 1000 or 20 μm respectively) from naïve animals (top panel), and from rats at 6 (second middle panel) and 24 h (third bottom panel) after bromine exposure. The black arrow in the middle panel indicates inflammatory cells and the black arrow in the third panel marks the tubular cast formation. The magnified square box demonstrates necrotic cells and epithelial cells shed in the tubular lumen marked by arrowheads in the inset. The lower panel displays images magnified at 40X from rats at 6 (fourth middle panel) and 24 h (bottom panel) after chlorine inhalation. The magnified square box in the fourth middle panel demonstrates necrotic cells and epithelial cells shed in the tubular lumen marked by arrowheads in the inset whereas the arrow indicates tubular cast formation. In the bottom panel the solid arrows indicate inflammatory cells, and the open arrows demonstrate the tubular casts in the renal tissues 24 h after exposure. The lower left image is a schematic representation of tubular epithelial cell death (B) followed by sloughing of the epithelium (C).

Histopathological analyses further corroborated our laboratory findings of kidney damage after bromine exposure. H&E staining of the kidney sections revealed interstitial damage with loss of normal tissue organization that progressed with time (Figure 4). Perivascular and interstitial edema with mononuclear infiltrate were observed in both chlorine and bromine exposed kidney sections (Figure 4 upper three panels and lower two panels). Higher resolution images demonstrated acute tubular epithelial injury with cell shedding (Inset to middle panel high resolution images). Epithelial flattening and casts and vacuoles were observed in the tubular lumen in the bromine exposed group at 24 h (Figure 4). Loss of glomerular contour was also observed in the kidneys of the bromine exposed animals at 24 h (Figure 4).

### 3.5 Bromine Inhalation Leads to Delayed Glomerular and Interstitial Fibrosis

**FIGURE 5:**
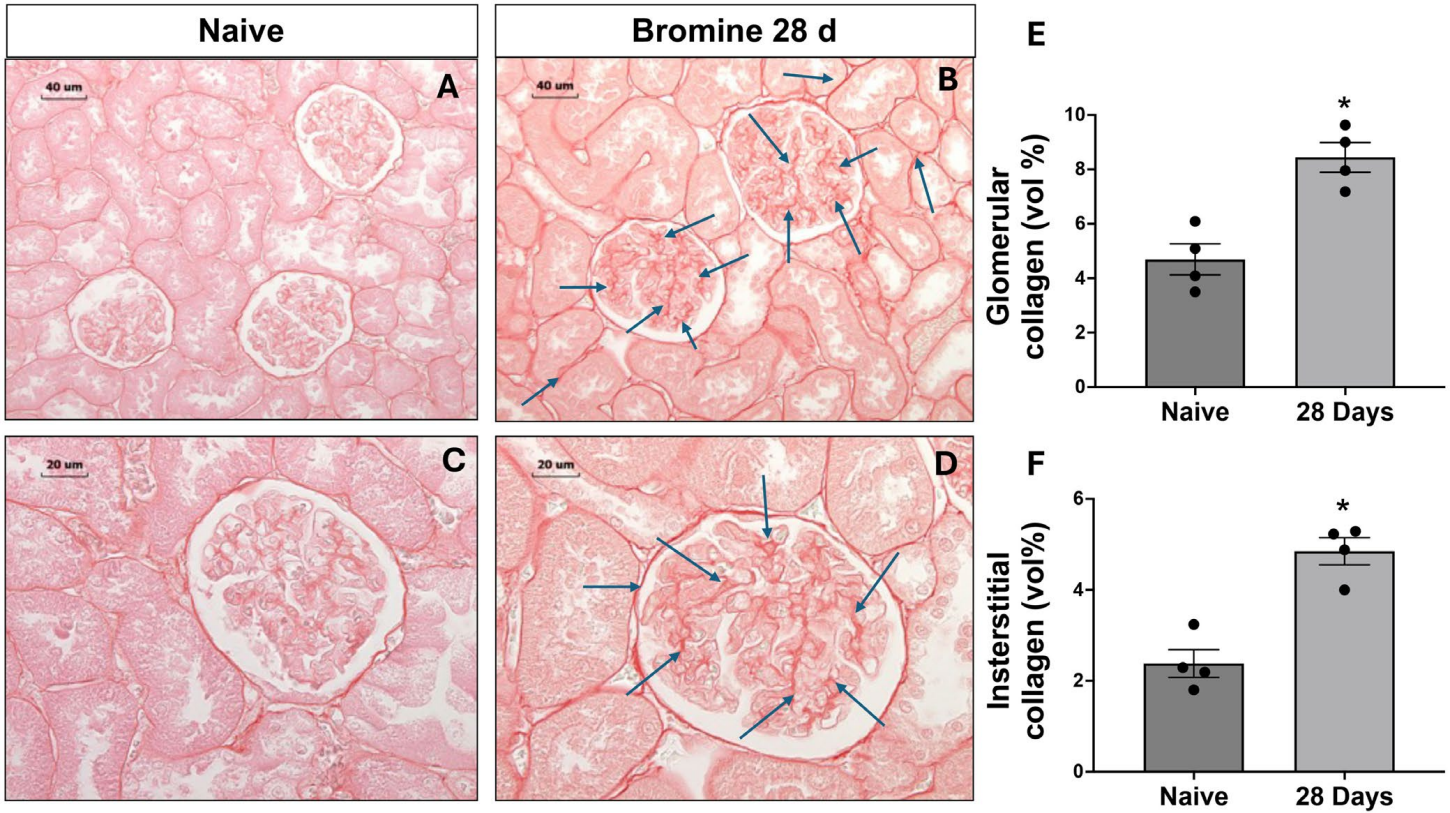
**Bromine inhalation leads to delayed glomerular and interstitial fibrosis.** Rats were exposed to bromine and necropsied 28 days post‐exposure, as described in Figure 1 and the Methods section. Kidneys were fixed, embedded, sectioned at 5 μm, and stained with picrosirius red (PSR) to assess collagen deposition. Panels A and B show representative low‐magnification PSR‐stained sections from naïve and bromine‐exposed rats, respectively; panels C and D show corresponding high‐magnification images. Quantification of glomerular collagen volume (%) is shown in panel E, and interstitial collagen volume (%) in panel F. Data are presented as mean ± SEM (*n* = 6–8). *p < 0.05 versus unexposed controls (naïve).

Acute kidney injury may progress to a more chronic form due to aberrant repair and interstitial fibrosis (Black et al. 2018; Grgic et al. 2012; Perez‐Moreno et al. 2025). To evaluate if halogen damaged kidneys develop fibrosis, we observed the bromine‐exposed animals for four weeks post‐exposure. Kidney sections of bromine‐exposed animals exhibited a significant increase in fibrosis as shown by the PASR staining of the kidney sections (Figure 5A–D). A significant increase in collagen volume was identified when measured either in the interstitium or in the glomerulus of the bromine‐exposed rats (Figure 5B,D and quantification in Figure 5E,F).

### 3.6 Oxidative Stress Contributes to Halogen (Cl_2_ or Br_2_)‐Induced Renal Injury

**FIGURE 6:**
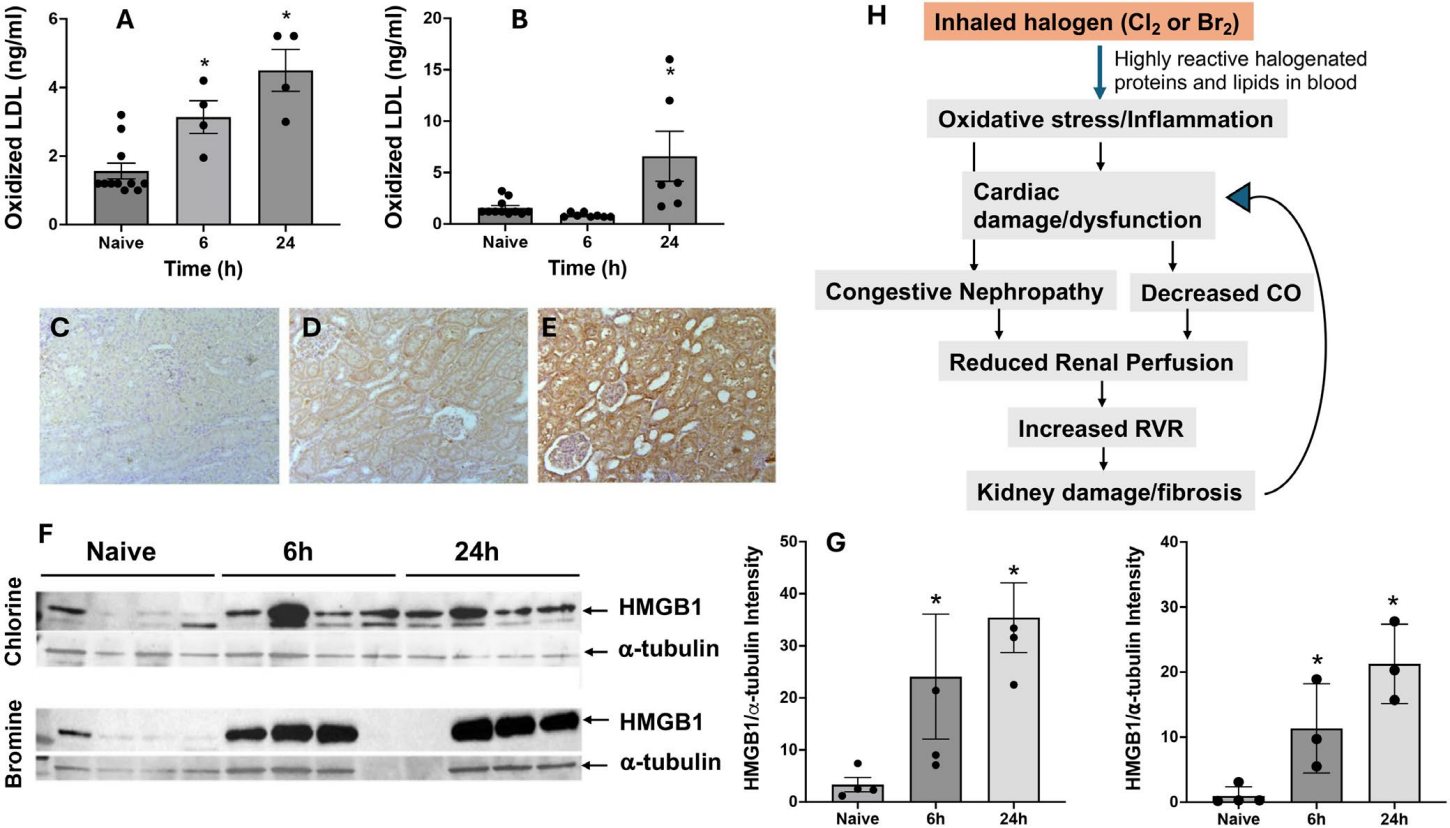
Oxidative stress contributes to halogen (Cl_2_ and Br_2_)‐induced renal injury. Rats were exposed to chlorine or bromine, returned to room air, and necropsied at 6‐ or 24‐h post‐exposure, as detailed in Figure 1 and the Methods section. Plasma samples from chlorine (A) and bromine (B) exposed rats were analyzed for oxidized LDL content. Kidney sections (5 μm) were immunostained for myeloperoxidase (MPO) using an anti‐rat MPO antibody followed by an HRP‐conjugated secondary antibody. Representative images are shown for naïve (C), 6 h post‐exposure (D), and 24 h post‐exposure (E). (F) Western blotting was performed to evaluate HMGB1 content in the renal tissues of chlorine (top panel) and bromine (bottom panel) exposed rats 6 h or 24 h after exposure. Quantitation of the blots are shown in (G left graph is for chlorine and right graph is for bromine). Data are presented as mean ± SEM (*n* = 6–8). *p < 0.05 versus unexposed controls (naïve). Panel H illustrates a schematic model of halogen‐induced renal injury via oxidative stress.

Myeloperoxidase is the primary source of oxidative stress in animals (Klebanoff and Rosen 1978). It is abundant in neutrophils, monocytes, and macrophages, and bromine inhalation increases neutrophil influx and myeloperoxidase (MPO) expression in the hearts of exposed animals (Ahmad, Masjoan Juncos, et al. 2019). MPO‐mediated lipid peroxidation and subsequent oxidative stress can induce kidney damage (Himmelfarb et al. 2001). Formation of oxidized low‐density lipoproteins (LDL) may cause detrimental effects to the kidney (Yang et al. 2023; Smith 2020). We found increased oxidized LDL content in the plasma of rats exposed to chlorine (Figure 6A) or bromine (Figure 6B). Immunohistochemistry was performed to evaluate the MPO content of the kidneys following bromine exposure and discovered enhanced MPO expression in the kidneys. The protein high mobility group box 1 (HMGB1) plays a pivotal role in regulating cellular responses to oxidative stress (Tang et al. 2011; Yu et al. 2015). HMGB1 is also a proinflammatory cytokine that damages the kidneys and other organs in AKI (Matsuura et al. 2023). Our studies with inhaled toxicants demonstrate an increase in bronchoalveolar lavage fluid HMGB1 upon lung injury (Manzoor et al. 2020; Mariappan et al. 2020; Mariappan et al. 2023; Ahmad, Zafar, et al. 2019). Additionally, our findings show that both chlorine and bromine increased the HMGB1 content in the kidneys of the exposed animals 6 h and 24 h post‐exposure (Figure 6F,G).

## 4 Discussion

Halogen gases like chlorine and bromine are increasingly being produced and used worldwide, raising the risk of occupational or accidental exposures. They are highly reactive, and inhalational injuries often lead to fatalities. Besides inducing pulmonary damage and ARDS, halogens have been shown to cause injury to distant organs such as the heart, vasculature, brain, and placenta. Renal damage and its mechanisms after halogen inhalation are unknown. In this manuscript, we describe that acute exposure to halogen gases (chlorine and bromine) increases markers of renal injury in the urine and blood, reduces renal blood flow, narrows renal artery diameter, and increases renal vascular resistance and mean arterial pressure. Histological assessment confirmed both interstitial and tubular damage with a loss of glomerular contour. Casts and dead cells were also found in the lumen of tubules, further providing evidence of structural damage. Increased inflammation and oxidative stress upon halogen exposure were found to be the mechanisms behind the acute kidney injury besides loss of perfusion.

Inhaled environmental and occupational halogen exposures are known to cause kidney damage, dysfunction, and the onset of chronic kidney diseases (Scammell et al. 2019; Tsai et al. 2021). Extrapulmonary distribution of inhaled particulate matter (PM 2.5) and subchronic exposure to ozone can lead to membranous glomerulonephritis and renal damage (Li et al. 2025; Yarbakht et al. 2025). Halogenated persistent organic pollutants (POPs) have been determined to accumulate in large amounts in feline kidneys and cause adverse renal health effects in cats (Nomiyama et al. 2024). Nephrotoxicity of halogenated anesthetics has been a cause for concern of anesthesiologists for decades (Reichle et al. 2002). Halogenated flame retardants pose a significant health risk for humans, and exposure to them may cause acute renal damage in preclinical models (Alharbi and Alhujaily 2024; Milovanovic et al. 2018; Soderlund et al. 1988; Sun et al. 2021). Our studies with bromine in rats and other studies with chlorine exposures in sheep demonstrated similar toxicity in both genders (Masjoan Juncos et al. 2021; Baljinnyam et al. 2023). Since both males and females seemed to have similar effects of halogen inhalation on blood creatinine in our studies, this susceptibility could be regardless of gender. Thus, it may be reasonably concluded that kidneys are susceptible to inhaled toxicants, particularly the halogenated molecules.

Plasma creatinine clearance has been associated with renal function for some time and is routinely used as a biomarker for assessment of kidney health (Losito 2023). However, creatinine bioassays in mouse and rat samples are often inaccurate due to interference of chromogens such as heme (Keppler et al. 2007). Although they provide an early estimate of creatinine values, the results must be considered with caution as halogen exposure causes RBC lysis and free heme generation (Aggarwal et al. 2020). A number of mass spectrometry‐based methods that quantify serum or plasma creatinine with great specificity are available; however, they require specific sample storage and preparation and there are no studies regarding interferences (Curtis et al. 2021; Ou et al. 2015). Our studies previously demonstrated that blood sampled from the coronary sinus of chlorine‐exposed rats had significantly increased creatinine content, which potentially reflects cardio‐renal interaction aside from cardiac myolysis (Zaky et al. 2015). Although renal effects of chlorine inhalation are largely unknown, inhalation of chlorine dioxide causes high levels of creatinine in the blood of exposed individuals (Bathina et al. 2013). Additionally, exposure to chlorinated insecticides increased creatinine and blood urea nitrogen (BUN) in affected animals, while brominated compounds used as flame retardants and disinfectants cause increased creatinine contents in circulation (Milovanovic et al. 2018; Omurtag et al. 2008; Tao et al. 2024).

Serum creatinine is an insensitive and nonspecific marker of kidney function and does not inform on the site, magnitude, or recovery of renal insults. Therefore we sought to explore additional evidence of renal injury in halogen exposed animals (Bolisetty and Agarwal 2011; Doi et al. 2009). Urine protein and albumin were shown to increase quickly upon renal damage caused by intrinsic factors such as ischemia reperfusion (Bolisetty and Agarwal 2011; Ware et al. 2011). Kidney‐derived proteins like retinol binding protein 4 (RBP4) reflect tubular damage when found in urine (Palviainen et al. 2012). Urinary retinol binding protein 4 (uRBP4) is the most sensitive biomarker for renal pathologies where tubular injury is a driving force (Ratajczyk et al. 2022; Norden et al. 2014). Our study expands the value of uRBP4 as a biomarker for inhaled chemical induced damage to the kidneys.

Neutrophil gelatinase activated lipocalin, NGAL, is one of the earliest and most robustly expressed proteins after ischemic or nephrotoxic kidney injury (Devarajan 2008). It is mainly produced by activated neutrophils, but also expressed in other cells including those of the kidneys and lungs (Romejko et al. 2023). NGAL, also known as lipocalin 2 (LCN2), is significantly increased in BALF of mice with bleomycin‐induced pulmonary fibrosis and in human IPF patients (Tanahashi et al. 2024). It is known to cause organ injury by promoting inflammation and oxidative stress (An et al. 2023; Wang et al. 2022). Kidney injury molecule‐1 (Kim‐1) is highly expressed in the proximal tubular region and sheds into the urine and blood upon injury (Sabbisetti et al. 2014). Increased osteopontin levels were also found to be strongly associated with acute tubular injury (Schmidt et al. 2024). Osteopontin released into the blood from kidneys following an injury was shown to cause acute lung injury and respiratory failure (Khamissi et al. 2022). This study emphasizes the importance of these biomarkers for identification and progression of inhaled halogen gas‐induced organ injury. Our finding suggests hypernatremia may be a marker associated with the development of AKI that could signify profound dehydration resulting from reduced renal blood flows or the beginning of renal injury due to the loss of urine dilution capacity (Zaragoza et al. 2024). The lack of hyperkalemia in our study despite the evidence of severe injury may indicate an early stage of renal failure that has not yet progressed to the final stage of total functional loss.

Renal blood flow is a major determinant of renal function (Beloncle et al. 2019). However, the relationship between renal blood flow, mean arterial pressure, and renal function is complex and relatively unexplored in a clinical context. Renal blood flow reduction could be a consequence of a decrease in cardiac output that may result from systolic dysfunction occurring due to halogen inhalation, as shown by our previous studies (Masjoan Juncos et al. 2021b; Masjoan Juncos et al. 2024; Ahmad, Masjoan Juncos, et al. 2019; Zaky et al. 2015). Additional neurohormonal mechanisms may also be involved, but reduced renal blood flow is a known indicator of kidney damage (Anand et al. 1991). Studies in this paper were performed with ultrasound of the ‘left’ renal artery, a common technique used by researchers to acquire clear and precise data for renal function assessment. Moreover, the right renal artery is often obscured by visceral organs such as the liver and spleen, which creates difficulty for images to be obtained of the right kidney (Balıkçı Dorotea et al. 2016). Because ultrasound is not sensitive enough to assess differences between blood flow from the left and right kidneys, and given the complexity of the assessment, we continued to use only the left renal artery measurements for consistency. While using more advanced imaging techniques (ASL MRI) in three different rat species, including those of the Sprague Dawley (used in this study) rats, it was observed that the renal blood flow was similar in both the left and right kidneys of naïve animals (Romero et al. 2018). This study demonstrates that toxic chemical inhalation‐induced AKI is associated with decreased renal perfusion as a consequence of the decreased renal artery diameter and blood flow and increased renal resistance and systemic mean arterial pressure.

Although the systemic pathophysiological changes that come from halogen inhalation resulting in pulmonary and distant organ damage are not very well understood, halogens do cause increased systemic inflammation and oxidative stress. Oxidative stress is a primary agent in the toxicity of both chlorine and bromine (Zhou et al. 2018). Plasma lipids and lipoproteins are susceptible to oxidative damage, and formation of oxidized HDL (Ox‐HDL) reduces HDL function that has been proposed to play a role in cardiorenal syndrome pathogenesis (Peterson et al. 2020). Additionally, Ox‐HDL readily converts to ox‐LDL which is cytotoxic (Alomar et al. 1992; Hurtado et al. 1996). Oxidized LDL was shown to increase progressively in diabetics leading to CKD and aggravating preexisting AKI (Roumeliotis et al. 2021; Yang et al. 2023; Sun et al. 2025). Inflammatory cells, especially neutrophils, infiltrate the lungs upon chlorine inhalation (Zarogiannis et al. 2011). Neutrophil‐derived myeloperoxidase (MPO) has a significant role in the oxidation of biomolecules such as low‐density lipoproteins and hence contributes to oxidative organ damage and disease pathogenesis (Kisic et al. 2016). The damage‐associated molecular pattern (DAMP) protein high mobility group box 1 (HMGB1) is pivotal in regulating cellular responses to oxidative stress (Tang et al. 2011; Yu et al. 2015). It is also a proinflammatory cytokine that causes damage to the kidneys and other organs in AKI (Matsuura et al. 2023). Due to its significant effect on the onset and progression of a number of diseases, it is gaining significant attention for potential druggability for clinical benefit (Datta et al. 2024). Therefore, our studies not only provide evidence of renal damage following halogen inhalation and describe the physiological impact and potential mechanisms for this damage but also propose potential therapeutic targets for alleviating the injury.

In this preclinical model, we demonstrate renal injury and dysfunction as a consequence of a more severe systemic inflammatory response to toxic inhalant exposure. The mechanism of renal injury is both primary and secondary. The primary mechanism is evidenced by urinary and tissue markers of injury. The secondary mechanism is likely caused by cardiac dysfunction and is manifested by a reduction in renal perfusion due to a decrease in cardiac output. A progressive pattern of renal damage is noticed by an increase in tissue markers of fibrosis. Yet the effects of renal damage on the heart remain to be studied. Determining cardiorenal reciprocity is clinically important to identify timely therapeutic targets that are likely to reverse and contain damage in both organs (Di Lullo et al. 2017). Furthermore, the progression of this acute form of renal injury to a more chronic form requires future exploration.

We describe a halogen inhalation model in rats where ischemic cardiac injury and acute renal injury occur concurrently. Clinically these data imply that a victim of halogen gas exposure is at risk of cardiovascular injury and renal dysfunction. Scenarios like this where two of the vital organs are damaged are common; however, the mechanisms of injury remain unknown. There is also a lack of animal models that replicate all such clinical features. Such approaches as this are needed to address major questions in the field, which include the diagnosis, prognosis, and treatment of both acute and chronic renal failure and the progression of acute kidney injury to chronic kidney disease.

## Author Contributions

Conceived and designed research, performed experiments: Juan Xavier Masjoan Juncos, Iram Zafar, Amber J. Johns, Wesam Nasser, Gajanan R. Jadhav, Aftab Ahmad, Shama Ahmad. Analyzed data: Juan Xavier Masjoan Juncos, Iram Zafar, Amber J. Johns, Wesam Nasser, Gajanan R. Jadhav, Shama Ahmad, Ahmed Zaky, Jeremy B. Foote. Interpreted results of experiments: Juan Xavier Masjoan Juncos, Iram Zafar, Amber J. Johns, Wesam Nasser, Gajanan R. Jadhav, Shama Ahmad, Jeremy B. Foote. Prepared figures: Juan Xavier Masjoan Juncos, Shama Ahmad, Jeremy B. Foote. Drafted manuscript: Juan Xavier Masjoan Juncos, Shama Ahmad, Aftab Ahmad, Anupam Agarwal. Edited and revised manuscript: Shama Ahmad, Aftab Ahmad, Anupam Agarwal, Ahmed Zaky. Approved final version of manuscript: Shama Ahmad, Aftab Ahmad, Anupam Agarwal, Ahmed Zaky.

## Funding

This work was funded by the CounterACT Program grants, National Institutes of Health Office of the Director (NIH OD), the National Institute of Environmental Health Sciences (NIEHS) Grants U01ES028182, U01ES033263, R21ES030525, R21ES032353, and R56ES034423.

## Conflicts of Interest

The authors declare no conflicts of interest.

## Supporting information


**Data S1:** cph470096‐sup‐0001‐DataS1.jpg.

**Figure S1:** Exposure to bromine causes increased blood creatinine content in male and female rats. Male or female rats were separately exposed to bromine (600 ppm for 45 min) and transferred to room air. Animals were sacrificed 24 h after exposure and blood from descending aorta was collected and analyzed for arterial blood gases (ABG analysis using a Heska EPOC machine) as described in the Methods section. (A) blood creatinine levels of bromine exposed rats post exposure, where filled symbols represent females and open symbols represent males. (B) serum creatinine levels of bromine exposed rats at various durations post exposure as measured by LC MS/MS. Values shown are Mean ± SEM (*n* = 6–8). * Indicates *p* < 0.05 as compared to unexposed controls (naives).
**Figure S2:** Exposure to bromine causes increased injury to kidney tissues. Bromine exposure was performed as described in the Methods section and above and animals were euthanized to collect the kidney tissues. Lysates were prepared and Western blotting was performed to evaluate the injury markers KIM1 and NGAL in the renal tissues (top panel) and the densitometric evaluation of the bands with respect to β‐actin loading control was also performed and shown in the lower panels. Values shown are Mean ± SEM (*n* = 4). * Indicates *p* < 0.05 as compared to unexposed controls (naives).
**Figure S3:** Chlorine and bromine inhalation induces acute structural kidney damage. Rats were exposed to chlorine or bromine, returned to room air, and euthanized at 72‐h post‐exposure, as described in the Methods section. Kidneys were fixed, embedded, sectioned at 5 μm, and stained with Jone's Silver Stain (Popov, Stoyanov, and Ghenev 2022). The top panel shows representative 40X magnification images of stained kidney sections from naïve animals, and from rats at 72 h after chlorine or bromine exposure. Green arrows indicate tubular epithelial cell shedding and red arrows demonstrate inflammatory cell accumulation.

## Data Availability

The data that support the findings of this study are available from the corresponding author upon reasonable request.

## References

[cph470096-bib-0001] Addis, D. R. , S. Aggarwal , A. Lazrak , T. Jilling , and S. Matalon . 2021. “Halogen‐Induced Chemical Injury to the Mammalian Cardiopulmonary Systems.” Physiology (Bethesda, Md.) 36: 272–291. 10.1152/physiol.00004.2021. 34431415 PMC8807065

[cph470096-bib-0002] Aggarwal, S. , A. Lam , S. Bolisetty , et al. 2016. “Heme Attenuation Ameliorates Irritant Gas Inhalation‐Induced Acute Lung Injury.” Antioxidants & Redox Signaling 24: 99–112. 10.1089/ars.2015.6347. 26376667 PMC4742996

[cph470096-bib-0003] Aggarwal, S. , A. Lazrak , I. Ahmad , et al. 2020. “Reactive Species Generated by Heme Impair Alveolar Epithelial Sodium Channel Function in Acute Respiratory Distress Syndrome.” Redox Biology 36: 101592. 10.1016/j.redox.2020.101592. 32506040 PMC7276446

[cph470096-bib-0004] Ahmad, S. , J. X. Masjoan Juncos , A. Ahmad , et al. 2019. “Bromine Inhalation Mimics Ischemia‐Reperfusion Cardiomyocyte Injury and Calpain Activation in Rats.” American Journal of Physiology. Heart and Circulatory Physiology 316: H212–H223. 10.1152/ajpheart.00652.2017. 30379573 PMC6383353

[cph470096-bib-0005] Ahmad, S. , I. Zafar , N. Mariappan , et al. 2019. “Acute Pulmonary Effects of Aerosolized Nicotine.” American Journal of Physiology. Lung Cellular and Molecular Physiology 316: L94–L104. 10.1152/ajplung.00564.2017. 30358437 PMC6383503

[cph470096-bib-0006] Alharbi, A. , and M. Alhujaily . 2024. “Molecular Mechanism of Indoor Exposure to Airborne Halogenated Flame Retardants TCIPP (Tris(1,3‐Dichloro‐2‐Propyl) Phosphate) and TCEP Tris(2‐Chloroethyl) Phosphate and Their Hazardous Effects on Biological Systems.” Metabolites 14: 120697. 10.3390/metabo14120697. PMC11677016 39728479

[cph470096-bib-0007] Alomar, Y. , A. Nègre‐Salvayre , T. Levade , P. Valdiguie , and R. Salvayre . 1992. “Oxidized HDL Are Much Less Cytotoxic to Lymphoblastoid Cells Than Oxidized LDL.” Biochimica et Biophysica Acta 1128, no. 2–3: 163–166. 1420286 10.1016/0005-2760(92)90302-c

[cph470096-bib-0008] An, H. S. , J. W. Yoo , J. H. Jeong , et al. 2023. “Lipocalin‐2 Promotes Acute Lung Inflammation and Oxidative Stress by Enhancing Macrophage Iron Accumulation.” International Journal of Biological Sciences 19: 1163–1177. 10.7150/ijbs.79915. 36923935 PMC10008694

[cph470096-bib-0009] Anand, I. S. , J. Gurden , G. S. Wander , et al. 1991. “Cardiovascular and Hormonal Effects of Calcitonin Gene‐Related Peptide in Congestive Heart Failure.” Journal of the American College of Cardiology 17: 208–217. 10.1016/0735-1097(91)90729-s. 1987228

[cph470096-bib-0010] Balıkçı Dorotea, S. , T. Banzato , L. Bellini , B. Contiero , and A. Zotti . 2016. “Kidney Measures in the Domestic Rat: A Radiographic Study and a Comparison to Ultrasonographic Reference Values.” Journal of Exotic Pet Medicine 25: 157–162. 10.1053/j.jepm.2016.03.011.

[cph470096-bib-0011] Baljinnyam, T. , Y. Niimi , J. R. Salsbury , et al. 2023. “Dose and Gender Dependence of Chlorine Inhalation in a Conscious Ovine Model.” Scientific Reports 13: 22367. 10.1038/s41598-023-48720-2. 38102196 PMC10724231

[cph470096-bib-0012] Bathina, G. , Y. Manjusha , B. Srikanth , et al. 2013. “An Unusual Case of Reversible Acute Kidney Injury due to Chlorine Dioxide Poisoning.” Renal Failure 35: 1176–1178. 10.3109/0886022X.2013.819711. 23902291

[cph470096-bib-0013] Beloncle, F. , L. Piquilloud , and P. Asfar . 2019. “Chapter 18—Renal Blood Flow and Perfusion Pressure.” In Critical Care Nephrology, edited by C. Ronco , R. Bellomo , and J. A. Kellum , 3rd ed., 106–109. Elsevier.

[cph470096-bib-0014] Black, L. M. , J. M. Lever , A. M. Traylor , et al. 2018. “Divergent Effects of AKI to CKD Models on Inflammation and Fibrosis.” American Journal of Physiology. Renal Physiology 315: F1107–F1118. 10.1152/ajprenal.00179.2018. 29897282 PMC6230746

[cph470096-bib-0015] Bolisetty, S. , and A. Agarwal . 2011. “Urine Albumin as a Biomarker in Acute Kidney Injury.” American Journal of Physiology. Renal Physiology 300: F626–F627. 10.1152/ajprenal.00004.2011. 21228105 PMC3064133

[cph470096-bib-0016] Curtis, L. M. , J. George , V. Vallon , et al. 2021. “UAB‐UCSD O'brien Center for Acute Kidney Injury Research.” American Journal of Physiology. Renal Physiology 320: F870–F882. 10.1152/ajprenal.00661.2020. 33779316 PMC8424552

[cph470096-bib-0017] Darriverre, L. , F. Fieux , and C. de la Jonquiere . 2020. “Acute Renal Failure During COVID‐19 Epidemic.” Praticien en Anesthesie Reanimation 24: 207–211. 10.1016/j.pratan.2020.07.004. 32837207 PMC7351375

[cph470096-bib-0018] Datta, S. , M. A. Rahman , S. Koka , and K. M. Boini . 2024. “High Mobility Group Box 1 (HMGB1): Molecular Signaling and Potential Therapeutic Strategies.” Cells 13: 13. 10.3390/cells13231946. PMC11639863 39682695

[cph470096-bib-0019] Devarajan, P. 2008. “Neutrophil Gelatinase‐Associated Lipocalin (NGAL): A New Marker of Kidney Disease.” Scandinavian Journal of Clinical and Laboratory Investigation. Supplementum 241: 89–94. 10.1080/00365510802150158. 18569973 PMC2528839

[cph470096-bib-0020] Di Lullo, L. , A. Bellasi , V. Barbera , et al. 2017. “Pathophysiology of the Cardio‐Renal Syndromes Types 1–5: An Upto Date.” Indian Heart Journal 69: 255–265. 28460776 10.1016/j.ihj.2017.01.005 PMC5415026

[cph470096-bib-0021] Doi, K. , P. S. Yuen , C. Eisner , et al. 2009. “Reduced Production of Creatinine Limits Its Use as Marker of Kidney Injury in Sepsis.” Journal of the American Society of Nephrology: JASN 20: 1217–1221. 10.1681/ASN.2008060617. 19389851 PMC2689892

[cph470096-bib-0022] Farha, N. , and C. Munguti . 2020. “A Dramatic Presentation of Pulmonary Edema due to Renal Failure.” Kansas Journal of Medicine 13: 56–57. 32226582 PMC7100944

[cph470096-bib-0023] Grgic, I. , G. Campanholle , V. Bijol , et al. 2012. “Targeted Proximal Tubule Injury Triggers Interstitial Fibrosis and Glomerulosclerosis.” Kidney International 82: 172–183. 10.1038/ki.2012.20. 22437410 PMC3480325

[cph470096-bib-0024] Himmelfarb, J. , M. E. McMenamin , G. Loseto , and J. W. Heinecke . 2001. “Myeloperoxidase‐Catalyzed 3‐Chlorotyrosine Formation in Dialysis Patients.” Free Radical Biology & Medicine 31: 1163–1169. 10.1016/s0891-5849(01)00697-9. 11705694

[cph470096-bib-0025] Hong, S. K. , D. K. Anestis , N. M. Hawco , M. A. Valentovic , P. I. Brown , and G. O. Rankin . 1996. “Nephrotoxicity of N‐(3‐Bromophenyl)‐2‐Hydroxysuccinimide: Role of Halogen Groups in the Nephrotoxic Potential of N‐(Halophenyl) Succinimides.” Toxicology 110: 17–25. 10.1016/0300-483x(96)03328-8. 8658556

[cph470096-bib-0026] Hurtado, I. , C. Fiol , V. Gracia , and P. Caldú . 1996. “In Vitro Oxidised HDL Exerts a Cytotoxic Effect on Macrophages.” Atherosclerosis 125: 39–46. 10.1016/0021-9150(96)05840-6. 8831925

[cph470096-bib-0027] Jankowski, J. , J. Floege , D. Fliser , M. Böhm , and N. Marx . 2021. “Cardiovascular Disease in Chronic Kidney Disease.” Circulation 143: 1157–1172. 10.1161/CIRCULATIONAHA.120.050686. 33720773 PMC7969169

[cph470096-bib-0028] Juncos, J. X. M. , S. Shakil , A. Ahmad , et al. 2020. “Circulating and Tissue Biomarkers as Predictors of Bromine Gas Inhalation.” Annals of the New York Academy of Sciences 1480: 104–115. 10.1111/nyas.14422. 32645215 PMC7708511

[cph470096-bib-0029] Keppler, A. , N. Gretz , R. Schmidt , et al. 2007. “Plasma Creatinine Determination in Mice and Rats: An Enzymatic Method Compares Favorably With a High‐Performance Liquid Chromatography Assay.” Kidney International 71: 74–78. 10.1038/sj.ki.5001988. 17082757

[cph470096-bib-0030] Khamissi, F. Z. , L. Ning , E. Kefaloyianni , et al. 2022. “Identification of Kidney Injury–Released Circulating Osteopontin as Causal Agent of Respiratory Failure.” Science Advances 8: eabm5900. 10.1126/sciadv.abm5900. 35213222 PMC8880785

[cph470096-bib-0031] Kisic, B. , D. Miric , I. Dragojevic , J. Rasic , and L. Popovic . 2016. “Role of Myeloperoxidase in Patients With Chronic Kidney Disease.” Oxidative Medicine and Cellular Longevity 2016: 1069743. 10.1155/2016/1069743. 27127544 PMC4834151

[cph470096-bib-0032] Klebanoff, S. J. , and H. Rosen . 1978. “The Role of Myeloperoxidase in the Microbicidal Activity of Polymorphonuclear Leukocytes.” Ciba Foundation Symposium: 263–284. 10.1002/9780470715413.ch15. 225142

[cph470096-bib-0033] Kubrak, T. , R. Podgórski , D. Aebisher , and A. Gala . 2018. “The Significance of NGAL and KIM‐1 Proteins for Diagnosis of Acute Kidney Injury (AKI) in Clinical Practice.” European Journal of Clinical and Experimental Medicine 16: 28–33. 10.15584/ejcem.2018.1.4.

[cph470096-bib-0034] Lag, M. , E. J. Soderlund , J. G. Omichinski , et al. 1991. “Effect of Bromine and Chlorine Positioning in the Induction of Renal and Testicular Toxicity by Halogenated Propanes.” Chemical Research in Toxicology 4: 528–534. 10.1021/tx00023a007. 1793801

[cph470096-bib-0035] Lam, A. , N. Vetal , S. Matalon , and S. Aggarwal . 2016. “Role of Heme in Bromine‐Induced Lung Injury.” Annals of the New York Academy of Sciences 1374: 105–110. 10.1111/nyas.13086. 27244263 PMC4940273

[cph470096-bib-0036] Lambert, J. A. , M. A. Carlisle , A. Lam , et al. 2017. “Mechanisms and Treatment of Halogen Inhalation‐Induced Pulmonary and Systemic Injuries in Pregnant Mice.” Hypertension 70: 390–400. 10.1161/HYPERTENSIONAHA.117.09466. 28607126 PMC5518712

[cph470096-bib-0037] Lazrak, A. , J. R. Creighton , Z. Yu , et al. 2015. “Hyaluronan Mediates Airway Hyper‐Responsiveness in Oxidative Lung Injury.” American Journal of Physiology. Lung Cellular and Molecular Physiology 308: 2014. 10.1152/ajplung.00377.2014. PMC4421785 25747964

[cph470096-bib-0038] Leikauf, G. D. , H. Pope‐Varsalona , V. J. Concel , et al. 2012. “Integrative Assessment of Chlorine‐Induced Acute Lung Injury in Mice.” American Journal of Respiratory Cell and Molecular Biology 47: 234–244. 10.1165/rcmb.2012-0026OC. 22447970 PMC3423464

[cph470096-bib-0039] Leustik, M. , S. Doran , A. Bracher , et al. 2008. “Mitigation of Chlorine‐Induced Lung Injury by Low‐Molecular‐Weight Antioxidants.” American Journal of Physiology. Lung Cellular and Molecular Physiology 295: L733–L743. 10.1152/ajplung.90240.2008. 18708632 PMC2584876

[cph470096-bib-0040] Li, M. , S. Chen , X. Jiang , et al. 2025. “Subchronic Ozone Exposure Leads to Multi‐Organ Injuries With Differential Reversibility in Male C57BL/6 J Mice.” Journal of Hazardous Materials 492: 138049. 10.1016/j.jhazmat.2025.138049. 40157190

[cph470096-bib-0041] Lindsey, M. L. , Z. Kassiri , J. A. I. Virag , L. E. de Castro Bras , and M. Scherrer‐Crosbie . 2018. “Guidelines for Measuring Cardiac Physiology in Mice.” American Journal of Physiology. Heart and Circulatory Physiology 314: H733–H752. 10.1152/ajpheart.00339.2017. 29351456 PMC5966769

[cph470096-bib-0042] Liubchenko, P. N. , and G. A. Alekseeva . 1991. “Acute Poisoning With Bromine Vapors of a Pharmaceutical Plant Operator.” Gigiena Truda i Professional'nye Zabolevaniia 9: 32–34. 1794718

[cph470096-bib-0043] Losito, A. 2023. “History of Creatinine Clearance: Tribute to a Forerunner.” Clinical Kidney Journal 16: 891–895. 10.1093/ckj/sfad024. 37638352 PMC10448966

[cph470096-bib-0044] Makarovsky, I. , G. Markel , A. Hoffman , et al. 2007. “Bromine—The Red Cloud Approaching.” Israel Medical Association Journal: IMAJ 9: 677–679. 17939634

[cph470096-bib-0045] Malek, M. , J. Hassanshahi , R. Fartootzadeh , F. Azizi , and S. Shahidani . 2018. “Nephrogenic Acute Respiratory Distress Syndrome: A Narrative Review on Pathophysiology and Treatment.” Chinese Journal of Traumatology 21: 4–10. 10.1016/j.cjtee.2017.07.004. 29398292 PMC5835491

[cph470096-bib-0046] Manzoor, S. , N. Mariappan , I. Zafar , et al. 2020. “Cutaneous Lewisite Exposure Causes Acute Lung Injury.” Annals of the New York Academy of Sciences 1479: 210–222. 10.1111/nyas.14346. 32329907 PMC8325512

[cph470096-bib-0047] Marahrens, B. , L. Damsch , R. Lehmann , I. Matyukhin , S. Patschan , and D. Patschan . 2023. “Increased Serum Sodium at Acute Kidney Injury Onset Predicts In‐Hospital Death.” Journal of Clinical Medical Research 15: 90–98. 10.14740/jocmr4845. PMC9990719 36895623

[cph470096-bib-0048] Mariappan, N. , M. Husain , I. Zafar , et al. 2020. “Extracellular Nucleic Acid Scavenging Rescues Rats From Sulfur Mustard Analog‐Induced Lung Injury and Mortality.” Archives of Toxicology 94: 1321–1334. 10.1007/s00204-020-02699-1. 32157350 PMC7230031

[cph470096-bib-0049] Mariappan, N. , I. Zafar , A. Robichaud , et al. 2023. “Pulmonary Pathogenesis in a Murine Model of Inhaled Arsenical Exposure.” Archives of Toxicology 97: 1847–1858. 10.1007/s00204-023-03503-6. 37166470 PMC11562768

[cph470096-bib-0050] Masjoan Juncos, J. X. , F. Nadeem , S. Shakil , et al. 2024. “Myocardial SERCA2 Protects Against Cardiac Damage and Dysfunction Caused by Inhaled Bromine.” Journal of Pharmacology and Experimental Therapeutics 390: 146–158. 10.1124/jpet.123.002084. 38772719 PMC11192580

[cph470096-bib-0051] Masjoan Juncos, J. X. , S. Shakil , A. Ahmad , et al. 2021a. “Sex Differences in Cardiopulmonary Effects of Acute Bromine Exposure.” Toxicology Research (Camb.) 10: 1064–1073. 10.1093/toxres/tfab079. PMC8557644 34733491

[cph470096-bib-0052] Masjoan Juncos, J. X. , S. Shakil , W. E. Bradley , et al. 2021b. “Chronic Cardiac Structural Damage, Diastolic and Systolic Dysfunction Following Acute Myocardial Injury due to Bromine Exposure in Rats.” Archives of Toxicology 95: 179–193. 10.1007/s00204-020-02919-8. 32979061 PMC7855670

[cph470096-bib-0053] Matsuura, R. , Y. Komaru , Y. Miyamoto , et al. 2023. “HMGB1 Is a Prognostic Factor for Mortality in Acute Kidney Injury Requiring Renal Replacement Therapy.” Blood Purification 52: 660–667. 10.1159/000530774. 37336200 PMC10614245

[cph470096-bib-0054] Milovanovic, V. , A. Buha , V. Matovic , et al. 2018. “Oxidative Stress and Renal Toxicity After Subacute Exposure to Decabrominated Diphenyl Ether in Wistar Rats.” Environmental Science and Pollution Research International 25: 7223–7230. 10.1007/s11356-015-5921-5. 26676538

[cph470096-bib-0055] Morabia, A. , C. Selleger , P. Conne , J. C. Landry , and J. Fabre . 1986. “Bromine Cloud in Geneva. Epidemiologic Study of the Short‐Term Effects on a Population Sample.” Schweizerische Medizinische Wochenschrift 116: 11–18. 3945787

[cph470096-bib-0056] Muto, S. , M. Imai , and Y. Asano . 1992. “Interaction of Cl‐ and Other Halogens With Cl‐ Transport Systems in Rabbit Cortical Collecting Duct.” American Journal of Physiology 263: F870–F877. 10.1152/ajprenal.1992.263.5.F870. 1443175

[cph470096-bib-0059] National Institutes of Health . 1996. “NTP Renal Toxicity Studies of Selected Halogenated Ethanes Administered by Gavage to F344/N Rats.” Toxicity Report Series 45: 1–C3. 11965399

[cph470096-bib-0057] Nomiyama, K. , R. Sato , F. Sato , and A. Eguchi . 2024. “Accumulation of Persistent Organic Pollutants in the Kidneys of Pet Cats ( *Felis silvestris* Catus) and the Potential Implications for Their Health.” Science of the Total Environment 933: 173212. 10.1016/j.scitotenv.2024.173212. 38759481

[cph470096-bib-0058] Norden, A. G. , M. Lapsley , and R. J. Unwin . 2014. “Urine Retinol‐Binding Protein 4: A Functional Biomarker of the Proximal Renal Tubule.” Advances in Clinical Chemistry 63: 85–122. 10.1016/b978-0-12-800094-6.00003-0. 24783352

[cph470096-bib-0060] Omurtag, G. Z. , A. Tozan , A. O. Sehirli , and G. Sener . 2008. “Melatonin Protects Against Endosulfan‐Induced Oxidative Tissue Damage in Rats.” Journal of Pineal Research 44: 432–438. 10.1111/j.1600-079X.2007.00546.x. 18205731

[cph470096-bib-0061] Ou, M. , Y. Song , S. Li , et al. 2015. “LC‐MS/MS Method for Serum Creatinine: Comparison With Enzymatic Method and Jaffe Method.” PLoS One 10: e0133912. 10.1371/journal.pone.0133912. 26207996 PMC4514740

[cph470096-bib-0062] Palviainen, M. , M. Raekallio , M. M. Rajamäki , J. Linden , and O. Vainio . 2012. “Kidney‐Derived Proteins in Urine as Biomarkers of Induced Acute Kidney Injury in Sheep.” Veterinary Journal 193: 287–289. 10.1016/j.tvjl.2011.10.004. 22088561

[cph470096-bib-0063] Perez‐Moreno, E. , A. de la Pena , T. Toledo , et al. 2025. “Endogenous Galectin‐8 Protects Against Th17 Infiltration and Fibrosis Following Acute Kidney Injury.” Molecular Medicine (Cambridge, Mass) 31: 192. 10.1186/s10020-025-01245-y. 40375122 PMC12083165

[cph470096-bib-0064] Peterson, S. J. , A. Choudhary , A. K. Kalsi , S. Zhao , R. Alex , and N. G. Abraham . 2020. “OX‐HDL: A Starring Role in Cardiorenal Syndrome and the Effects of Heme Oxygenase‐1 Intervention.” Diagnostics (Basel) 10: 10. 10.3390/diagnostics10110976. PMC7699797 33233550

[cph470096-bib-0065] Radbel, J. , D. L. Laskin , J. D. Laskin , and H. M. Kipen . 2020. “Disease‐Modifying Treatment of Chemical Threat Agent‐Induced Acute Lung Injury.” Annals of the New York Academy of Sciences 1480: 14–29. 10.1111/nyas.14438. 32726497 PMC10250775

[cph470096-bib-0066] Ratajczyk, K. , A. Konieczny , A. Czekaj , et al. 2022. “The Clinical Significance of Urinary Retinol‐Binding Protein 4: A Review.” International Journal of Environmental Research and Public Health 19: 169878. 10.3390/ijerph19169878. PMC9408023 36011513

[cph470096-bib-0067] Reichle, F. M. , P. F. Conzen , and K. Peter . 2002. “Nephrotoxicity of Halogenated Inhalational Anaesthetics: Fictions and Facts.” European Surgical Research 34: 188–195. 10.1159/000048908. 11867922

[cph470096-bib-0068] Romejko, K. , M. Markowska , and S. Niemczyk . 2023. “The Review of Current Knowledge on Neutrophil Gelatinase‐Associated Lipocalin (NGAL).” International Journal of Molecular Sciences 24: 1310470. 10.3390/ijms241310470. PMC10341718 37445650

[cph470096-bib-0069] Romero, C. A. , G. Cabral , R. A. Knight , G. Ding , E. L. Peterson , and O. A. Carretero . 2018. “Noninvasive Measurement of Renal Blood Flow by Magnetic Resonance Imaging in Rats.” American Journal of Physiology. Renal Physiology 314: F99–f106. 10.1152/ajprenal.00332.2017. 28978533 PMC5866354

[cph470096-bib-0070] Roumeliotis, S. , A. Roumeliotis , P. I. Georgianos , et al. 2021. “Oxidized LDL Is Associated With eGFR Decline in Proteinuric Diabetic Kidney Disease: A Cohort Study.” Oxidative Medicine and Cellular Longevity 2021: 2968869. 10.1155/2021/2968869. 34712380 PMC8548137

[cph470096-bib-0071] Sabbisetti, V. S. , S. S. Waikar , D. J. Antoine , et al. 2014. “Blood Kidney Injury Molecule‐1 Is a Biomarker of Acute and Chronic Kidney Injury and Predicts Progression to ESRD in Type I Diabetes.” Journal of the American Society of Nephrology 25: 2177–2186. 10.1681/asn.2013070758. 24904085 PMC4178434

[cph470096-bib-0072] Scammell, M. K. , C. M. Sennett , Z. E. Petropoulos , J. Kamal , and J. S. Kaufman . 2019. “Environmental and Occupational Exposures in Kidney Disease.” Seminars in Nephrology 39: 230–243. 10.1016/j.semnephrol.2019.02.001. 31054622 PMC12881994

[cph470096-bib-0073] Schmidt, I. M. , A. L. Surapaneni , R. Zhao , et al. 2024. “Plasma Proteomics of Acute Tubular Injury.” Nature Communications 15: 7368. 10.1038/s41467-024-51304-x. PMC11349760 39191768

[cph470096-bib-0074] Seeley, E. J. 2013. “Updates in the Management of Acute Lung Injury: A Focus on the Overlap Between AKI and ARDS.” Advances in Chronic Kidney Disease 20: 14–20. 10.1053/j.ackd.2012.10.001. 23265592

[cph470096-bib-0075] Smith, L. E. 2020. “High‐Density Lipoproteins and Acute Kidney Injury.” Seminars in Nephrology 40: 232–242. 10.1016/j.semnephrol.2020.01.013. 32303285

[cph470096-bib-0076] Soderlund, E. J. , J. G. Omichinski , J. E. Dahl , S. D. Nelson , and E. Dybing . 1988. “Nephrotoxicity of Selectively Deuterated and Methylated Analogues of Tris‐BP and Bis‐BP in the Rat.” Pharmacology & Toxicology 62: 142–149. 10.1111/j.1600-0773.1988.tb01862.x. 3375186

[cph470096-bib-0077] Summerhill, E. M. , G. W. Hoyle , S. E. Jordt , et al. 2017. “An Official American Thoracic Society Workshop Report: Chemical Inhalational Disasters. Biology of Lung Injury, Development of Novel Therapeutics, and Medical Preparedness.” Annals of the American Thoracic Society 14: 1060–1072. 10.1513/AnnalsATS.201704-297WS. 28418689 PMC5529138

[cph470096-bib-0078] Sun, D. Q. , M. Y. Zhong , J. H. Zhang , et al. 2025. “Oxidized‐LDL Aggravates Renal Injury via Tubular Cuproptosis.” Cellular Signalling 132: 111839. 10.1016/j.cellsig.2025.111839. 40306349

[cph470096-bib-0079] Sun, S. , Y. Jin , J. Yang , Z. Zhao , and Q. Rao . 2021. “Nephrotoxicity and Possible Mechanisms of Decabrominated Diphenyl Ethers (BDE‐209) Exposure to Kidney in Broilers.” Ecotoxicology and Environmental Safety 208: 111638. 10.1016/j.ecoenv.2020.111638. 33396158

[cph470096-bib-0080] Tanahashi, H. , H. Iwamoto , K. Yamaguchi , et al. 2024. “Lipocalin‐2 as a Prognostic Marker in Patients With Acute Exacerbation of Idiopathic Pulmonary Fibrosis.” Respiratory Research 25: 195. 10.1186/s12931-024-02825-y. 38704585 PMC11070072

[cph470096-bib-0081] Tang, D. , R. Kang , H. J. Zeh , and M. T. Lotze . 2011. “High‐Mobility Group Box 1, Oxidative Stress, and Disease.” Antioxidants & Redox Signaling 14: 1315–1335. 10.1089/ars.2010.3356. 20969478 PMC3048826

[cph470096-bib-0082] Tao, W. , W. Nian , and L. Li . 2024. “Analysis of Brominated Flame Retardants Exposure‐Associated Chronic Kidney Disease Risk in the US Population From the NHANES.” Ecotoxicology and Environmental Safety 286: 117159. 10.1016/j.ecoenv.2024.117159. 39383822

[cph470096-bib-0083] Tsai, H. J. , P. Y. Wu , J. C. Huang , and S. C. Chen . 2021. “Environmental Pollution and Chronic Kidney Disease.” International Journal of Medical Sciences 18: 1121–1129. 10.7150/ijms.51594. 33526971 PMC7847614

[cph470096-bib-0084] Valentovic, M. A. , J. G. Ball , D. K. Anestis , et al. 1992. “Acute Renal and Hepatic Toxicity of 2‐Haloanilines in Fischer 344 Rats.” Toxicology 75: 121–131. 10.1016/0300-483x(92)90151-4. 1462350

[cph470096-bib-0085] Wang, X. , C. Zhang , N. Zou , et al. 2022. “Lipocalin‐2 Silencing Suppresses Inflammation and Oxidative Stress of Acute Respiratory Distress Syndrome by Ferroptosis via Inhibition of MAPK/ERK Pathway in Neonatal Mice.” Bioengineered 13: 508–520. 10.1080/21655979.2021.2009970. 34969358 PMC8805876

[cph470096-bib-0086] Ware, L. B. , A. C. Johnson , and R. A. Zager . 2011. “Renal Cortical Albumin Gene Induction and Urinary Albumin Excretion in Response to Acute Kidney Injury.” American Journal of Physiology. Renal Physiology 300: F628–F638. 10.1152/ajprenal.00654.2010. 21147844 PMC3064135

[cph470096-bib-0087] Yang, D. , X. Yang , S. Chen , M. Lv , J. Tan , and D. Yang . 2023. “Ox‐LDL Aggravates Contrast‐Induced Injury of Renal Tubular Epithelial Cells.” Journal of Biochemical and Molecular Toxicology 37: e23379. 10.1002/jbt.23379. 37186061

[cph470096-bib-0088] Yarbakht, M. , G. Sarau , Y. Xu , et al. 2025. “Fine Particulate Matter (PM(2.5)) Induces microRNA‐192‐5p Causing Glomerular Damage.” Ecotoxicology and Environmental Safety 298: 118280. 10.1016/j.ecoenv.2025.118280. 40373708

[cph470096-bib-0089] Yu, Y. , D. Tang , and R. Kang . 2015. “Oxidative Stress‐Mediated HMGB1 Biology.” Frontiers in Physiology 6: 93. 10.3389/fphys.2015.00093. 25904867 PMC4387954

[cph470096-bib-0090] Zaky, A. , W. E. Bradley , A. Lazrak , et al. 2015. “Chlorine Inhalation‐Induced Myocardial Depression and Failure.” Physiological Reports 3: 12439. 10.14814/phy2.12439. PMC4510636 26109193

[cph470096-bib-0091] Zaragoza, J. J. , J. A. Gómez‐Fregoso , E. M. Hernández‐Barajas , et al. 2024. “Acute Kidney Injury With Hypernatremia and Major Adverse Kidney Events.” Clinical Kidney Journal 18: 419. 10.1093/ckj/sfae419. PMC12086689 40390839

[cph470096-bib-0092] Zarogiannis, S. G. , A. Jurkuvenaite , S. Fernandez , et al. 2011. “Ascorbate and Deferoxamine Administration After Chlorine Exposure Decrease Mortality and Lung Injury in Mice.” American Journal of Respiratory Cell and Molecular Biology 45: 386–392. 10.1165/rcmb.2010-0432OC. 21131440 PMC3175564

[cph470096-bib-0093] Zellner, T. , and F. Eyer . 2020. “Choking Agents and Chlorine Gas—History, Pathophysiology, Clinical Effects and Treatment.” Toxicology Letters 320: 73–79. 10.1016/j.toxlet.2019.12.005. 31811912

[cph470096-bib-0094] Zhang, J. , P. Zhu , S. Li , Y. Gao , and Y. Xing . 2023. “From Heart Failure and Kidney Dysfunction to Cardiorenal Syndrome: TMAO May Be a Bridge.” Frontiers in Pharmacology 14: 1291922. 10.3389/fphar.2023.1291922. 38074146 PMC10703173

[cph470096-bib-0095] Zhou, T. , W. F. Song , Y. Shang , S. L. Yao , and S. Matalon . 2018. “Halogen Inhalation‐Induced Lung Injury and Acute Respiratory Distress Syndrome.” Chinese Medical Journal 131: 1214–1219. 10.4103/0366-6999.231515. 29722341 PMC5956773

[cph470096-bib-0096] Zoccali, C. , F. Mallamaci , J. M. Halimi , et al. 2024. “From Cardiorenal Syndrome to Chronic Cardiovascular and Kidney Disorder: A Conceptual Transition.” Clinical Journal of the American Society of Nephrology 19: 813–820. 10.2215/cjn.0000000000000361. 37902772 PMC11168830

