# Supplementary figures and images for: Inhaled Halogen‐Induced Oxidative Renal Damage and Dysfunction: A Lung Heart Kidney Axis
Source: Compr Physiol. 2026 Jan 6;16(1):e70096. doi: 10.1002/cph4.70096 (PMC12775725; doi:10.1002/cph4.70096)

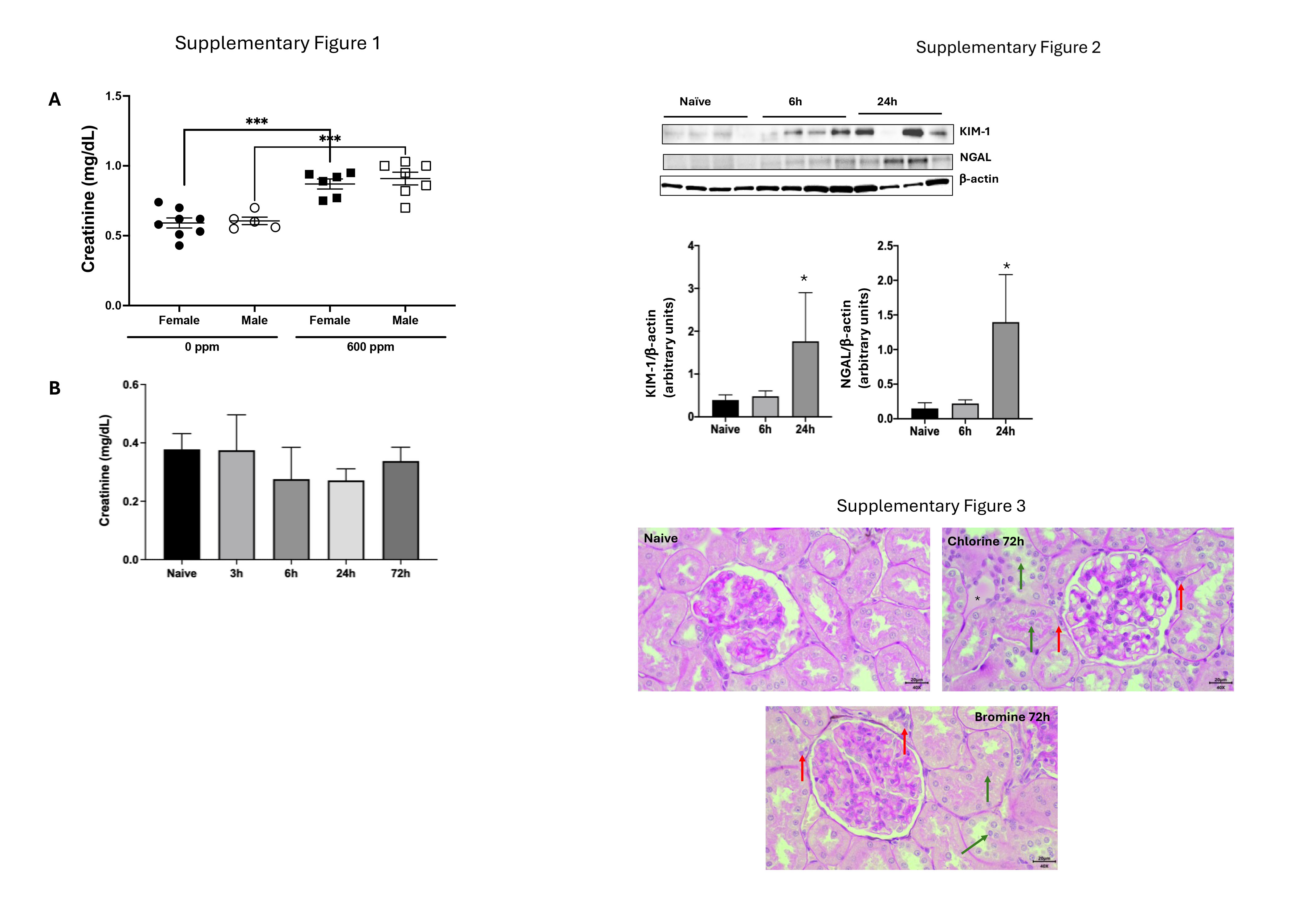

Supplement: Supplementary file 1 — Data S1: cph470096‐sup‐0001‐DataS1.jpg. [file CPH4-16-e70096-s001.jpg]
