# Supplementary material for: Inhaled Halogen‐Induced Oxidative Renal Damage and Dysfunction: A Lung Heart Kidney Axis
Source: Compr Physiol. 2026 Jan 6;16(1):e70096. doi: 10.1002/cph4.70096 (PMC12775725; doi:10.1002/cph4.70096)
Supplement: Supplementary file 2 — Figure S1: Exposure to bromine causes increased blood creatinine content in male and female rats. Male or female rats were separately exposed to bromine (600 ppm for 45 min) and transferred to room air. Animals were sacrificed 24 h after exposure and blood from descending aorta was collected and analyzed for arterial blood gases (ABG analysis using a Heska EPOC machine) as described in the Methods section. (A) blood creatinine levels of bromine exposed rats post exposure, where filled symbols represent females and open symbols represent males. (B) serum creatinine levels of bromine exposed rats at various durations post exposure as measured by LC MS/MS. Values shown are Mean ± SEM (n = 6–8). * Indicates p < 0.05 as compared to unexposed controls (naives). Figure S2: Exposure to bromine causes increased injury to kidney tissues. Bromine exposure was performed as described in the Methods section and above and animals were euthanized to collect the kidney tissues. Lysates were prepared and Western blotting was performed to evaluate the injury markers KIM1 and NGAL in the renal tissues (top panel) and the densitometric evaluation of the bands with respect to β‐actin loading control was also performed and shown in the lower panels. Values shown are Mean ± SEM (n = 4). * Indicates p < 0.05 as compared to unexposed controls (naives). Figure S3: Chlorine and bromine inhalation induces acute structural kidney damage. Rats were exposed to chlorine or bromine, returned to room air, and euthanized at 72‐h post‐exposure, as described in the Methods section. Kidneys were fixed, embedded, sectioned at 5 μm, and stained with Jone's Silver Stain (Popov, Stoyanov, and Ghenev 2022). The top panel shows representative 40X magnification images of stained kidney sections from naïve animals, and from rats at 72 h after chlorine or bromine exposure. Green arrows indicate tubular epithelial cell shedding and red arrows demonstrate inflammatory cell accumulation. [file CPH4-16-e70096-s002.docx]

Inhaled halogen-induced oxidative renal damage and dysfunction; a lung heart kidney axis

Juan Xavier Masjoan Juncos^1^, Ahmed Zaky^1^, Wesam Nasser^1^, Amber J Johns, Iram Zafar^1^, Gajanan R Jadhav^1^, Aftab Ahmad^1^, Anupam Agarwal^2^, Shama Ahmad^1*^.

^1^Department of Anesthesiology and Perioperative Medicine, University of Alabama at Birmingham, Birmingham, Al;

^2^Department of Medicine, Division of Nephrology, University of Alabama at Birmingham, Birmingham, Al;

**Legends to Supplementary Figures**

**Supplementary Figure 1. Exposure to bromine causes increased blood creatinine content in male and female rats.** Male or female rats were separately exposed to bromine (600 ppm for 45 min) and transferred to room air. Animals were sacrificed 24h after exposure and blood from descending aorta was collected and analyzed for arterial blood gases (ABG analysis using a Heska EPOC machine) as described in the Methods section. A) blood creatinine levels of chlorine exposed rats post exposure, where filled symbols represent females and open symbols represent males. B) serum creatinine levels of bromine exposed rats at various durations post exposure as measured by LC MS/MS. Values shown are Mean±SEM (n=6-8). * Indicates p<0.05 as compared to unexposed controls (naives).

**Supplementary Figure 2. Exposure to bromine causes increased injury to kidney tissues.** Bromine exposure was performed as described in the Methods section and above and animals were euthanized to collect the kidney tissues. Lysates were prepared and Western blotting was performed to evaluate the injury markers KIM1 and NGAL in the renal tissues (top panel) and the densitometric evaluation of the bands with respect to β-actin loading control was also performed and shown in the lower panels. Values shown are Mean±SEM (n=4). * Indicates p<0.05 as compared to unexposed controls (naives).

**Supplementary Figure 3. Chlorine and bromine inhalation induces acute structural kidney damage.** Rats were exposed to chlorine or bromine, returned to room air, and euthanized at 72-hours post-exposure, as described in the Methods section. Kidneys were fixed, embedded, sectioned at 5 μm, and stained with Jone’s Silver Stain (Popov, Stoyanov, and Ghenev 2022). The top panel shows representative 40X magnification images of stained kidney sections from naïve animals, and from rats at 72h after chlorine or bromine exposure. Green arrows indicate tubular epithelial cell shedding and red arrows demonstrate inflammatory cell accumulation.

**References:**

Popov, H., G. S. Stoyanov, and P. Ghenev. 2022. 'Modified Silver Impregnation Method for Basal Membranes in Renal Biopsies', *Cureus*, 14: e30171.
